# Polymorphous Adenocarcinoma: A Systematic Review of the Literature and Presentation of Two Cases in a Less-Considered Anatomical Site

**DOI:** 10.3390/cancers16010220

**Published:** 2024-01-03

**Authors:** Rodolfo Mauceri, Martina Coppini, Giuseppe Alecci, Adriana Cordova, Ada Maria Florena, Gaetano Magro, Corrado Toro, Giuseppina Campisi

**Affiliations:** 1 Department of Surgical, Oncological and Oral Sciences, University of Palermo, 90127 Palermo, Italy; rodolfo.mauceri@unipa.it (R.M.); giuseppina.campisi@policlinico.pa.it (G.C.); 2 Unit of Oral Medicine and Dentistry for Fragile Patients, Department of Rehabilitation, Fragility, and Continuity of Care, University Hospital Palermo, 90127 Palermo, Italy; 3 Department of Biomedical and Dental Sciences and Morphofunctional Imaging, University of Messina, 90122 Messina, Italy; 4 Department of Health Promotion Sciences, Maternal and Infant Care, Internal Medicine and Medical Specialties (ProMISE), University of Palermo, 90127 Palermo, Italy; giuseppe.alecci@community.unipa.it (G.A.); adamaria.florena@unipa.it (A.M.F.); 5 Plastic and Reconstructive Surgery Section, Department of Surgical, Oncological and Oral Sciences, University of Palermo, 90127 Palermo, Italy; adriana.cordova@unipa.it; 6 Department of Biomedical and Biotechnological Sciences, Section of Physiology, School of Medicine, University of Catania, 95123 Catania, Italy; gmagro@unict.it; 7 Maxillofacial Surgery Unit, Clinica del Mediterraneo of Ragusa, 97100 Ragusa, Italy; corradotoro@hotmail.com

**Keywords:** polymorphous adenocarcinoma, PAC, polymorphous low-grade adenocarcinoma, PLGA, salivary gland neoplasms, minor salivary glands, mouth, buccal mucosa

## Abstract

**Simple Summary:**

Polymorphous adenocarcinoma (PAC), the second most prevalent malignant tumour of the minor salivary glands, primarily affects the palate but can also involve the buccal mucosa. This systematic review investigates PAC’s clinical characteristics, emphasizing its presence in less-explored anatomical sites. Following PRISMA guidelines, we conducted a thorough search across PubMed, Scopus, and Web of Science, identifying and analysing 29 relevant studies. Among the 143 PAC patients included in the systematic review (62 males, 75 females, and 6 undefined, with a mean age of 57.4 ± 14.5 years), the palate was the predominant site affected by PAC (69.2%), followed by the buccal mucosa (8.4%). Additionally, we report two cases of PAC affecting the buccal mucosa, describing the mean features of an uncommon malignant neoplasm of the salivary glands. These findings underscore the significance of recognizing the buccal mucosa as a potential PAC site, urging careful examination during oral assessments due to the polymorphism and lack of pathognomonic signs.

**Abstract:**

Background: Polymorphous adenocarcinoma (PAC) is the second-most common malignant tumour of the minor salivary glands. Although PAC predominantly affects the palate, it can also involve the buccal mucosa. This systematic review aims to investigate the literature data about PAC. Furthermore, we report two cases of patients affected by PAC in an infrequently considered anatomical site. Methods: According to PRISMA guidelines, a systematic review search was conducted on PubMed, Scopus, and Web of Science. Observational studies conducted on patients with a histological diagnosis of PAC were selected and analysed. Furthermore, two cases of patients with PAC affecting the buccal mucosa were reported. Results: Twenty-nine studies were included, and 143 patients affected by PAC were analysed (62 males, 75 females, and 6 undefined, with a mean age of 57.4 ± 14.5 years). The palate was the most affected site (99/143, 69.2%), followed by the buccal mucosa (12/143, 8.4%). Moreover, we report two cases of patients with PAC affecting the buccal mucosa (one male and one female, with a mean age of 70.5 ± 2.5 years). Conclusions: The present study underscores the importance of considering the buccal mucosa as a possible location of minor salivary gland tumours; although it is a less-considered affliction, it is not uncommon.

## 1. Introduction

Polymorphous adenocarcinoma (PAC), previously known as polymorphous low-grade adenocarcinoma (PLGA), is an infrequent malignant neoplasm of the salivary glands [1].

PAC was first described in 1983 by Batsakis et al. as terminal duct carcinoma based on its originating from the terminal intercalary ducts and by Freedman and Lumerman as lobular carcinoma due to its resemblance to lobular breast carcinoma [2,3]. Subsequently, in 1984, the term “PLGA” was introduced to describe an infiltrative salivary tumour with a variety of growth patterns but bland nuclei [4]. Finally, in 2017, the fourth edition of the World Health Organization’s classification of salivary gland tumours replaced the term PLGA with polymorphous adenocarcinoma [5].

The term “polymorphous” describes the various architectural patterns that may be observed in PAC cases.

PAC is the second most common intraoral salivary gland malignancy; it predominantly affects the minor salivary glands of the palate but may also occur in the major salivary glands and extra-palatal sites [1].

Regarding the prevalence of anatomical sites affected by PAC, although the buccal mucosa is a less-considered site, it is not uncommon. PAC is usually diagnosed in the fifth decade of an individual’s life and is more common in females compared to males, with a ratio of 2:1 [6]. Typically, it manifests as mucosal swelling, and most patients remain asymptomatic. Common differential diagnoses include adenoid cystic carcinoma, pleomorphic adenoma, oral squamous cell carcinoma, and other benign or malignant salivary gland neoplasms [7]. Due to its morphological diversity and the absence of pathognomonic features, diagnosing PAC only through physical examination remains a challenge. Furthermore, since PAC often does not present as an exophytic lesion but instead preserves the overlying epithelium, it would be opportune to perform a meticulous examination of buccal mucosa during oral cavity screening. The diagnosis of clinical suspicion is usually supported by a biopsy with subsequent immunohistochemical analysis. To date, there is no gold standard for the treatment of PAC, even if surgical resection, at times combined with adjuvant radiation therapy, is preferred [8].

This systematic review investigates the main clinical features and global epidemiological trends of patients affected by PAC. Moreover, two cases of PAC affecting the buccal mucosa are discussed.

## 2. Materials and Methods

### 2.1. Systematic Review

#### 2.1.1. Protocol

A systematic literature search was conducted independently by two of this paper’s authors (MC and RM). The protocol for this study was designed following the Preferred Reporting Items for Systematic Reviews and Meta-Analyses (PRISMA) guidelines [9].

#### 2.1.2. PICo and Research Question

The research question was designed based on the PICo item, consisting of the following information:

P: Patients affected by PAC

I: Diagnosis of PAC

Co: Worldwide

The systematic review was based on the following research question: “What are the clinical characteristics and global epidemiological trends of patients affected by PAC worldwide?”

#### 2.1.3. Data Sources and Search Strategy

The selection of studies concerning patients affected by PAC with a histologically confirmed diagnosis was performed. Records were identified using different search engines (e.g., Medline/PubMed, Scopus, and Web of Science) and by scanning reference lists of articles. For the search strategy, MeSH terms and free-text words were combined through Boolean operators as follows: “mouth” AND (“Polymorphous LOW-GRADE adenocarcinoma” OR “PLGA” OR “Polymorphous adenocarcinoma” OR “PAC”). The research was completed in June 2023.

#### 2.1.4. Eligibility Criteria

The inclusion criteria for the studies were as follows:

- Human studies
- English language
- Studies reporting a histologically confirmed diagnosis of PAC

These criteria were selected to ensure a focused and standardized approach to data inclusion was followed. Human studies were prioritized to maintain clinical relevance, and the requirement for research presented in the English language ensured consistency in data interpretation. The strict inclusion of histologically confirmed PAC diagnoses was intended to enhance the precision and reliability of the study outcomes. In establishing these criteria, careful consideration was given to ensure the selection of studies that could provide robust and reliable data relevant to the objectives of the study and their reproducibility.

The exclusion criteria were as follows: studies focused on tumours different from PAC, narrative and systematic reviews and meta-analyses, and studies concerning patients affected by polymorphous low-grade adenocarcinoma in different sites (e.g., breast, pulmonary, etc.).

#### 2.1.5. Study Selection and Data Collection Processes

**Figure 1:**
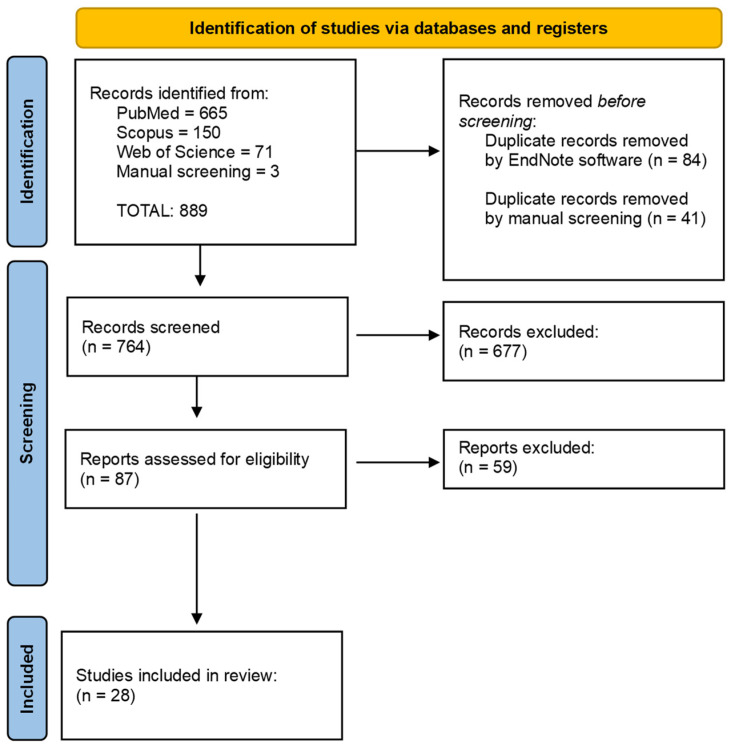
Flowchart of the different phases of the systematic review.

The initial search identified 889 records, of which 125 were removed as they were duplicates. The screening of 764 studies was performed based on the titles and abstracts, and 677 records were excluded. Subsequently, a full-text evaluation of 87 studies was carried out. Finally, based on the inclusion criteria, 59 records were excluded, and 28 papers were included in the current review; a detailed flow chart of the selection process is provided in Figure 1.

#### 2.1.6. Statistical Analysis

The selected studies were analysed to detect outcomes of interest. For each study, the following data were extracted using a pre-designed data extraction Excel sheet.

The following parameters were collected:

 i. Study characteristics: the name of the first author, the year of publication, the name of the country where the study was performed, and the design of the study.
ii. Main characteristics of the included patients: mean age, sex, and anatomical site of PAC.

Data extraction and descriptive analysis were performed using Microsoft Excel.

### 2.2. Case Series

The study protocol conformed to the ethical guidelines of the 1964 Declaration of Helsinki and its later amendments or comparable ethical standards. It was also approved by the Institutional Local Ethics Committee of the University Hospital “P. Giaccone” of Palermo, Palermo, Italy (approval #3/2013). All patients signed written informed consent.

#### 2.2.1. Data Collection and Clinical Examination

The clinical procedures were performed by two expert clinicians (GC and CT) at the Unit of Oral Medicine “V. Margiotta” of the University Hospital “Paolo Giaccone” in Palermo (Italy) and the Clinica del Mediterraneo in Ragusa (Italy). During the interview, variables including sociodemographic data, medical history, and a previous diagnosis of cancer were recorded. To assess health-related variables, the patients were interviewed concerning their current and past smoking history and alcohol consumption.

#### 2.2.2. Sample Collection

In the presence of a diagnostic suspicion of a neoplasm with uncertain behaviour affecting the oral cavity, an incisional biopsy was performed. Then, after local anaesthesia, an incisional biopsy was performed using a scalpel punch. Specimens were fixed in formalin solution and sent to the pathology laboratory for histopathological examination.

#### 2.2.3. Histological Examination and Immunohistochemistry

A microscopic evaluation was performed by two oral pathologists (AMF and GM). Histological examination was performed as follows: tissue sections were fixed in 10% buffered formalin and processed using routine histological techniques, including periodic acid-Schiff staining, alcian blue staining, and mucicarmine staining. Immunohistochemical studies were performed on formalin-fixed paraffin-embedded material using a streptavidin–biotin detection method.

## 3. Results

### 3.1. Systematic Review

Of the 87 total records assessed for eligibility, 28 were selected since they satisfied the inclusion criteria. The main characteristics of the included studies are reported in Table 1. All the included articles were observational studies published between 1984 and 2021. In detail, 8 articles were case series [4,10,11,12,13,14,15,16], and 20 were case reports [7,17,18,19,20,21,22,23,24,25,26,27,28,29,30,31,32,33,34,35]. Of the 28 studies, 8 were from India [7,25,27,30,31,32,33,34], 5 were from Spain [10,11,14,21,28], 3 were from Japan [13,26,35], 3 were from the USA [4,15,17], 1 was from Ireland [18], 1 was from Greece [19], 1 was from Israel [20], 1 was from the UK [12], 1 was from Italy [22], 1 was from Brazil [23], 1 was from Chile [24], 1 was from Korea [29], and 1 was from Denmark [16].

In total, 143 patients affected by PAC were analysed, of which 62 were males and 75 were females. Only one study did not specify the sex of the six included patients [14].

Based on the available data (except those presented by Fife et al. and Elhakim et al.), the mean age of patients affected by PAC was 57.4 ± 14.5 years. The average age of patients affected by PAC ranged from 16 to 86 years.

The age of the patients afflicted with PAC affecting the buccal mucosa ranged from 27 to 60 years, with a mean age of 44.2 ±11 years [4,18,20,31].

The palate was the site most affected by PAC (99/143, 69.2%), followed by the buccal mucosa (12/143, 8.4%), retromolar trigone (5/143, 3.5%), lip (8/143, 5.6%), tongue (3/143, 2.1%), parotid gland (6/143, 4.2%), pharynx (3/143, 2.1%), floor of the mouth (2/143, 1.4%), upper gingiva (1/143, 0.7%), sublingual gland (1/143, 0.7%), submandibular gland (1/143, 0.7%), maxillary sinus (1/143, 0.7%), and nose (1/143, 0.7%).

### 3.2. Case Reports

**Figure 2:**
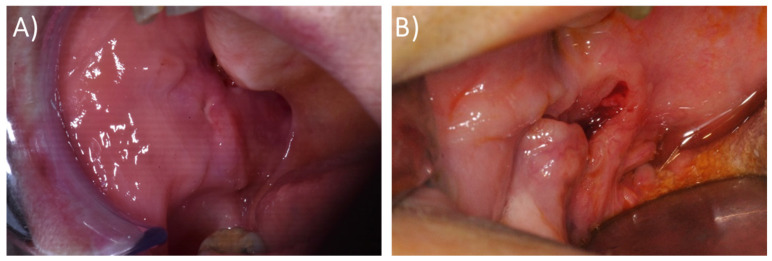
Clinical features at the first visit of case #1 (**A**) and case #2 (**B**).

**Figure 3:**
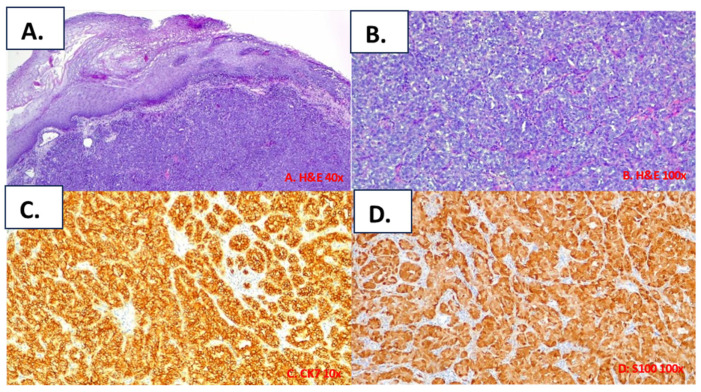
Histological features of case #1. Histological examination revealed the typical characteristics of a PAC of the conventional subtype: an infiltrative tumour in the sub-epithelial connective tissue (**A**), comprising uniform neoplastic cells arranged predominantly in a trabecular pattern (**B**); neoplastic cells showing diffuse positivity for cytokeratin 7 (**C**) and S100 protein (**D**).

**Figure 4:**
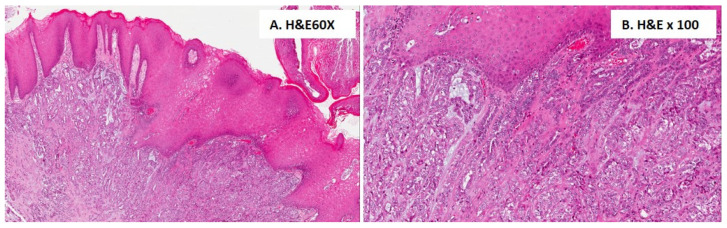
Histological features of case #2. Histological examination revealed the typical features of a PAC of the conventional subtype: an infiltrative tumour composed of uniform neoplastic cells arranged in both tubular and solid patterns within a fibrous stroma (**A**); neoplastic cells are closely adjacent to the squamous epithelium layer (**B**).

The main characteristics of the patients afflicted with PAC affecting the buccal mucosa included in the present study are summarized in Table 2. The clinical presentations of the patients included in this study are reported in Figure 2, and their histological features are reported in Figure 3 and Figure 4. In the following, the characteristics of the individual cases are reported.

*Case #1*

A 73-year-old Caucasian woman presented with a painless swelling of the right buccal mucosa. Her personal, medical, and surgical history did not include relevant information. She had no history of smoking or drinking. Physical examination revealed a submucosal nodule without surface ulceration on the right buccal mucosa; the nodule was not associated with pain (Figure 2A). An incisional biopsy was performed, and a diagnosis of PAC of the minor salivary glands was established. The patient was referred to the Oncology Unit for further management. PAC was surgically treated, and nodal metastasis was found via latero-cervical nodal dissection. Subsequently, the patient was subjected to radiotherapy. Currently, the patient has been in follow-up for 18 months without presenting recurrence.

*Case #2*

A 68-year-old Caucasian man presented with a painless submucosal mass on the right buccal mucosa. He was a smoker, and his personal medical and surgical history did not include relevant information. Physical examination revealed a submucosal nodule on the right buccal mucosa that did not present surface ulceration and was not associated with pain (Figure 2B). After confirming a histological diagnosis of PAC, a surgical resection was performed. Four years later, he is alive with no evidence of recurrence.

### 3.3. Histological Findings for the Included Cases

All tumours exhibited the typical features of polymorphous adenocarcinoma of the conventional subtype: infiltrative tumours composed of uniform neoplastic cells variably arranged in tubular, trabecular, and solid patterns within a fibrous stroma (Figure 3 and Figure 4). Most areas of the tumours showed pushing borders, whereas some exhibited infiltrative margins. The neoplastic cells were uniform in shape, with scant cytoplasm, bland nuclei, and often open chromatin. No necrosis was identified, and mitotic figures were infrequent. In one case (case#2), focal perineural invasion was documented (Figure 4). Immunohistochemically, the tumour was positive for epithelial markers, including anti-CK7 (Figure 3C), and was partially positive for p63; however, the tumour was negative for p40. The tumour cells showed diffuse immunoreactivity for S-100 (Figure 3D) and SOX10 proteins, but no immunostaining was observed for smooth muscle actin (alpha-SMA) and KIT (CD117). The MIB-1 (Ki-67) LI of the tumour cells was about 12%.

## 4. Discussion

Although the most frequent tumour affecting the oral cavity is OSCC, malignant salivary gland tumours account for 1–6% of head and neck cancers [36,37,38,39]. Minor salivary gland tumours are estimated to account for about 9–23% of all salivary gland neoplasms [37].

Even if minor salivary gland tumours occur relatively infrequently, they should not be undervalued because, unfortunately, about 80% of tumours of the minor glands are malignant [40].

Although PAC predominantly affects the minor salivary glands of the palate, it is not uncommon for it to involve the buccal mucosa.

In the present study, a systematic literature review was performed to investigate the most common clinical features and epidemiologic trends of PAC. Furthermore, we reported two cases of patients affected by PAC occurring in a less reported but not uncommon anatomical site.

PAC is a malignant salivary gland tumour that predominantly presents as an indolent, slow-growing submucosal mass in the oral cavity. In the initial stages, mucosal ulceration is rare, highlighting the challenge of diagnosing this condition in the early stages [8].

Specifically, regarding patients afflicted with PAC affecting the buccal mucosa, the common clinical presentation was asymptomatic swelling that remained quiescent for a long time before undergoing a rapid increase in size, leading to increasing discomfort [18,20].

Concerning the risk factors of PAC, a causal correlation was not observed in the reported cases. Known risk factors for salivary gland carcinoma include tobacco and alcohol use, a history of radiotherapy administered to the head and neck region, and HIV infection [41]. Regarding the potential role of oral microbiota in the carcinogenesis process, very few studies have investigated the salivary microbiota composition of patients afflicted with salivary gland tumours. In contrast to the established association of certain types of microorganisms with carcinogenesis (e.g., *Helicobacter pylori* and gastric cancer, *Human Papilloma Virus* and cervical cancer, and *Salmonella typhi* and gallbladder cancer), an association between oral microbiota and oral cancer has not yet been completely demonstrated [42]. The causal relationship between microbiota and cancer is complex since the anatomy of the oral cavity is different from that of other body sites. The oral cavity has mucosal surfaces (the tongue, the buccal mucosa, the gingiva, and the palate), hard tissues (the teeth), and exocrine gland tissue (major and minor salivary glands), all of which present unique features with respect to microbiota composition [43]. The relationship between microbiota and carcinogenesis may be associated with chronic inflammatory processes, direct antiapoptotic effects, the activation of cell proliferation, the promotion of cellular invasion, and the production of carcinogenic metabolites [42,43].

Concerning the incidence of PAC, the average age of patients included in the systematic review ranged from 16 to 86 years, with a mean age of 57.4 ± 14.5 years. Furthermore, a higher prevalence among females was observed, suggesting a gender predisposition for PAC. Notably, according to data from the literature, this condition tends to manifest at a slightly younger age in women compared to that for men. Additionally, despite the prevalence of PAC in elderly patients, some cases of PAC in adolescents and children have been reported, emphasizing the importance of vigilance across diverse age groups [25,44].

The average age of our patients affected by PAC ranged from 62 to 73 years, with a mean age of 67.7 ± 4.5 years. There was no difference regarding male/female prevalence. Regarding clinical presentation, both patients presented with a painless submucosal mass without alterations in the overlying mucosa.

Regarding the anatomical site most affected by PAC, our study confirmed that the palate was the predominantly affected site. However, it also highlighted the buccal mucosa as the second-most-affected site. Thus, although the occurrence of PAC in the buccal mucosa is less reported, it is not uncommon [45].

The term “polymorphous adenocarcinoma” was coined to emphasize that the most striking feature of this tumour is its extreme morphological variability, consisting of the coexistence of different architectural patterns, including solid, trabecular, tubular, papillary, and cribriform patterns [46].

High-grade transformation can rarely occur in the form of severe nuclear atypia, prominent nucleoli, a high mitotic count, and tumour necrosis [47].

Due to its architectural diversity and lack of pathognomonic signs, it can be very difficult to distinguish PAC through a physical examination alone. As PAC may exhibit some overlapping features with adenoid cystic carcinoma, pleomorphic adenoma, myoepithelial carcinoma, and metastatic carcinoma, immunohistochemistry is considered essential in the diagnosis of PAC [48]. Typically, PAC is diffusely and strongly positive for S100, SOX10, CAM 5.2, and CK7. The Ki-67 proliferation index in this regard is typically less than 10%, although exceptions have been reported.

Several epithelial and myoepithelial markers are considered valuable diagnostic markers for PAC, but no consistent immunohistochemical profile has yet been identified [48].

Recently, several studies have reported that PAC usually has a p63-positive/p40-negative immune profile. Notably, the degree of p63 positivity is quite variable and can be focal/weak or diffuse and strong. P63 is normally detected in basal stem cells of squamous epithelia as well as in basal/myoepithelial cells in the breasts, sweat glands, prostate, and salivary glands [49]. P63 protein plays an essential role in the morphogenesis of the epidermis and limbs, in addition to acting as a transcription factor in the growth and development of many epithelial organs. P40, an isotype of p63, was used as a more specific marker of basal and myoepithelial cell differentiation. It was suggested that a p63/p40 immunohistochemical panel could be a useful tool for differentiating salivary gland tumours containing myoepithelial cells (p63+/p40+), such as pleomorphic adenoma and adenoid cystic carcinoma, from PAC, a well-known tumour lacking myoepithelial cells (p63+/p40−) [50].

Therefore, P53, p63, and p73 are three genes that play important roles in regulating the cell cycle and in apoptosis after DNA damage, in stem cell identification, and in cellular differentiation and that are expressed in basal and myoepithelial cells. While p53 is the most frequently mutated gene in human tumours, p63 and p73 are rarely mutated or deleted in cancers. Many studies have reported p63/p73 overexpression in human cancers, while others have shown that a loss of p63/p73 is associated with tumour progression and metastasis. Thus, whether p63 or p73 is a tumour suppressor gene or an oncogene has been a matter of debate [51]. Furthermore, one study reported that the expression of p63 and p73 is maintained in both benign and malignant salivary gland tumours with basaloid or myoepithelial differentiation, with p63 being a more specific marker of myoepithelial differentiation than p73 [52].

The fifth edition of the WHO Classification of Head and Neck Tumours emphasizes the importance of genomic and genetic alterations in many salivary gland tumours and includes recurrent molecular alterations in the definitions of many salivary gland tumours, including PAC [53].

Cribriform adenocarcinoma of salivary gland origin (CASG) is considered a distinctive subtype of PAC, characterized by a multinodular growth pattern separated by fibrous septa, a relatively uniform solid cribriform and microcystic architecture, and optically clear nuclei. CASG is associated with a propensity for being situated at the base of the tongue and a higher risk of lymph node metastasis [53].

The detection of a PRKD1 p.Glu710Asp hotspot mutation or the translocation of one of the PRKD1, PRKD2, or PRKD3 genes is now considered highly specific for the diagnosis of PAC, as these molecular alterations are rarely detected in other salivary gland tumours. The conventional subtype of PAC more frequently harbours a PRKD1 p.Glu710Asp hotspot mutation, as PRKD1/2/3 gene fusions are more frequently found in CASG.

Several authors have proposed considering CASG a separate entity from PAC based on its histological features, characteristic anatomic site, higher risk of lymph node metastasis, and peculiar cytogenetic signature [54].

The detection of these molecular alterations is now included in the desirable diagnostic criteria of PAC, although in selected cases, the morphological and immunophenotypical features of these tumours can be sufficient for diagnosis [53].

Regarding the treatment of PAC, to date, there is no gold standard [55]. To ensure the completeness of the study, a narrative review of possible treatments for PAC was also conducted.

Independently of the location of PAC, its primary management consists of complete surgical excision. Since metastases of neck lymph nodes are rarely reported, neck dissection should generally be performed only in the case of positive lymph nodes.

The roles of radiotherapy (RT) and chemotherapy are still controversial [56]. Different authors have reported that RT is most often used postoperatively, to treat patients with extensive primary tumours or when section margins are not clear, when there is perivascular or perineural spread ahead of the main front in the resected specimen, and/or when cervical nodal metastases have been found [57,58].

In patients without lymph node metastases, postoperative RT does not seem to influence the prognosis [27].

Regarding chemotherapy, although there is no specific evidence in the literature supporting its use in PAC treatment, some authors reported the use of chemotherapy restricted to palliative cases [57]. It may be used concomitantly with RT for unresectable PAC, when the patient refuses surgery is inoperable or in the postoperative setting [59,60].

PAC generally has a good prognosis after complete surgical excision, with a recurrence rate of about 15%, usually occurring within 5 to 7 years of treatment of the primary tumour, and recurrences are more common in women than in men [61].

Nevertheless, cases of recurrence have also been observed after 14 years [15]. For this reason, long-term follow-ups as long as 15 to 20 years have been suggested.

According to some studies, it seems that this entity can behave aggressively when it presents early in life, especially during adolescence [25].

Local recurrence and lymph node metastases were observed in several cases of PAC localized in the palate [4,11,14,25,26].

Although it may appear that localization in the palate may correlate with a poor prognosis, Seethala et al. reported that cancer originating from the base of the tongue frequently metastasizes toward cervical lymph nodes, suggesting the importance of the prevention of neck dissection for these patients [62].

Regarding the location of the tumour, the greater tendency of tumours located at the base of the tongue to metastasize could be related to their anatomical characteristics, such as their vascularization and the proximity to the cervical lymph nodes [63,64]. Therefore, depth of invasion is an important prognostic factor [65].

Even the palate exhibits peculiar anatomical features, characterized by an abundance of minor salivary glands and mucosa closely interconnected with the underlying periosteum. This distinctive composition gives rise to a greater spectrum of malignancies compared to that in other oral sites [66].

This study possesses some limitations derived from the small sample size, which was probably due to the rarity of the pathology investigated. Although PAC is not a common head and neck tumour, understanding and identifying the clinical features of PAC are essential for enhancing early diagnosis and treatment.

In summary, our study emphasizes the importance of not underestimating the buccal mucosa as an anatomical site for salivary malignant tumours and considering PAC in the differential diagnosis of fixed, firm, painless masses with intact overlying mucosa.

## 5. Conclusions

The present study emphasizes the significance of recognizing the buccal mucosa as a potential site for minor salivary gland tumours. Despite being less frequently considered, our systematic review highlights the occurrence of PAC in this anatomical location. The challenges posed by the absence of pathognomonic signs and the polymorphic nature of PAC make early diagnosis difficult through physical examination alone. Consequently, understanding the primary clinical characteristics and identifying the most prevalent anatomical locations of PAC are crucial. This knowledge can facilitate early diagnosis, improving prognosis and reducing potential complications. In addition to conducting a systematic review, we presented two cases of PAC affecting this less common anatomical site to underscore the necessity of evaluating the buccal mucosa as a potential site for malignant tumours.

## Figures and Tables

**Table 1:** The characteristics of the studies included in the literature review.

N.	Author, Year	Country	N. of Case	Age	Sex	Site
1	Evans HL, 1984 [4]	USA	14	74 (27–76)	8 M/6 F	11 palate; 2 buccal mucosa; 1 posterior mandible
2	Kennedy LKS, 1987 [17]	USA	1	48	M	tongue
3	Scally CM, 1988 [18]	Ireland	1	39	F	buccal mucosa
4	Nicolatou O, 1988 [19]	Greece	1	68	M	palate
5	Fliss DM, 1989 [20]	Israel	1	45	M	buccal mucosa
6	Colmenero CM, 1992 [10]	Spain	4	59.7 ± 16.3	1 M/3 F	3 palate; 1 retromolar pad
7	Dean A, 1994 [11]	Spain	2	53.5 ± 10.5	M	2 palate
8	Crean SJ, 1996 [12]	UK	4	52.5 ± 10.5	2 M/2 F	3 palate; 1 right retromolar area
9	De Diego JI, 1996 [21]	Spain	1	60	M	tongue
10	Mincione GP, 1999 [22]	Italy	1	67	M	soft palate
11	Nagao T, 2004 [13]	Japan	3	65 ± 11.4	2 M/1 F	2 parotid gland; 1 submandibular gland
12	Tincani AJ, 2005 [23]	Brazil	1	69	M	tongue
13	González-García R, 2005 [14]	Spain	6	61.2 ± 9.4	n.d.	palate
14	Pintor MF, 2007 [24]	Chile	1	65	F	hard palate and the alveolar ridge
15	Arora SK, 2013 [25]	India	1	18	M	hard palate
16	Kawahara A, 2013 [26]	Japan	1	70	M	palate
17	Gupta S, 2011 [27]	India	1	52	M	palate
18	Andreu-Barasoain M, 2013 [28]	Spain	1	75	F	upper lip
19	Lee DH, 2013 [29]	Korea	1	59	M	maxillary sinus
20	Fife TA, 2013 [15]	USA	17	62.7 ± 14.2	7 M/10 F	hard palate 7/17 (41.2%) hard/soft palate junction 5/17 (29.4%) soft palate 2/17 (11.8%) lip 2/17 (11.8%) retromolar trigone 1/17 (5.9%)
21	Tomar R 2015 [7]	India	1	45	M	upper lip
22	Radhika T, 2015 [30]	India	1	59	M	retromolar region
23	Elhakim MT, 2016 [16]	Denmark	73	58 (16–86)	26 M/47 F	3 parotid; 4 lip; 7 buccal mucosa; 2 floor of mouth; 53 palate; 1 nose; 3 pharynx
24	Sathyanarayanan R, 2015 [31]	India	1	63	F	palate
25	Khosla D, 2017 [32]	India	1	16	M	parotid gland
26	Vadla P, 2018 [33]	India	1	50	M	right upper posterior part of alveolus (upper gingiva)
27	Muniswammappa S, 2022 [34]	India	1	50	F	buccal mucosa
28	Nakasone T, 2021 [35]	Japan	1	81	F	sublingual gland

**Table 2:** Main characteristics of patients affected by PAC on the buccal mucosa reported in the present study.

Case	Age	Sex	Smoker	Surgical Treatment	Radiotherapy	Follow-Up (Months)	Recurrence/ Metastases
#1	73	F	No	Yes	No	18	Yes, lymph node metastases
#2	68	M	Yes	Yes	No	50	No

## Data Availability

Data sharing not applicable.
